# Review of the utilization of HEEPF – competitive projects for educational enhancement in the Egyptian medical sector

**DOI:** 10.1186/1478-4491-6-7

**Published:** 2008-04-18

**Authors:** Galal Abdel-Hamid Abdellah, Salah El-Din Mohamed Fahmy Taher, Somaya Hosny

**Affiliations:** 1 HEEPF National Committee, Egypt; 2 Higher Education Enhancement Project Fund, HEEPF, Egypt; 3 Suez Canal University, Egypt

## Abstract

In Egypt, the medical sector has been facing the same problems that challenged the system of higher education in the past decades, mainly an increasing student enrollment, limited resources, and old governance and bylaws. These constraints and the escalating paucity of resources have had a major negative influence on quality of education. Consequently, thoughts of educational reform came forward in the form of competitive projects, which have attracted several institutes from the health sector to improve their educational performance. The aim of this paper is to review the share of the medical sector in the higher education enhancement project fund (HEEPF), its outcomes, sustainability, and to provide recommendations for keeping the momentum of reform pursuit in the future. The methodology included obtaining statistics pertaining to the medical sector in Egypt as regards colleges, students, and staff. We also reviewed the self-studies of the medical sector colleges, HEEPF projects reports, performance appraisal reports, and World Bank reports on HEEPF achievements in order to retrieve the required data. Results showed that medical sector had a large share of the HEEPF (28.5% of projects) as compared to its size (8% of student population). The projects covered 10 areas; the frequency distribution of which ranged between 4.4% (creation of new programs) to 97.8% (human resource development). In conclusion, educational enhancement in the medical sector in Egypt could be apparently achieved through the HEEPF competitive projects. A study of the long-term impact of these projects on the quality of education is recommended

## Review

In Egypt, the medical sector of higher education includes five major specialties; medicine, pharmacy, dentistry, nursing, and physical medicine. It represents about 8% and 36% of the total force of higher education in terms of students enrolled and working staff, respectively. Thus, this sector has an average student teacher ratio of 9.5:1, which is a relatively better ratio as compared to the average total ratio in all sectors (39.8:1) [1].

Enrolment into higher education in Egypt depends solely on the total score of the secondary school graduation certificate. Applicants for medical sector colleges always have the highest marks among high school graduates, as these colleges admit relatively limited numbers of students in comparison to other sectors. Medical sector colleges have still a highly prestigious outlook and corroborate certified opportunities at national and regional job markets; this renders them a target for the highest achieving students [2].

Education in the medical sector is offered via three relatively independent bodies; public secular, public religious, and private. The public secular body contains 49 colleges in which 116 326 (79.7%) students are enrolled. The private body includes 13 colleges in which 19 942 (13.7%) students are registered, while the religious body (Al-Azhar University) has 10 colleges which have a total of 9601 (6.6%) students, according to data of academic year 2004–2005 [1].

**Figure 1:**
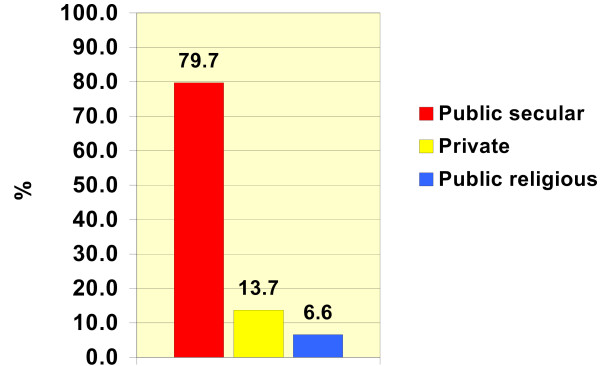
Distribution of students' enrolment in the three bodies of the medical Sector (Academic year 2004/2005).

Because the public secular body of the medical sector has the largest share in terms of students' enrolment (see Figure 1), it became the main target of the educational enhancement projects funded by the World Bank and the Egyptian government.

### Problems and challenges facing education in the medical sector

The system of higher education in Egypt has been facing a number of problems and challenges during the last decades. This situation that led to a state of low system efficiency was previously addressed and analyzed [3,4].

Besides the typical problems of overcrowding, limited financial resources, lack of a sustainable financial policy, inadequate infrastructure, under-trained faculty members in some areas, poor instructional materials and equipment and lack of a formal evaluation and accreditation mechanism, a number of other problems have emerged in the era of rapid explosion of information and modern communication technology. This latter group of problems can be summarized as follows:

• The enormous advances in information/communication technology (ICT) and its application to all activities including the teaching/learning process and academic administration constitute a major challenge to higher education due to the emerging need for changing the way of developing and delivering educational materials and services. It has also created a continuous need to update ICT and associated skills, and an increasing need for distance learning. Furthermore, incoming students increasingly have high ICT competencies, and consequently expect consistent ICT facilities in their higher education.

• The continuing emergence of new discoveries adding to the core of knowledge calls for a parallel enhancement of all aspects of relevant competencies of workforce, including knowledge, attitude and skills. Also pertinent here is keeping abreast with changes in how knowledge is certified.

• Increased competition with greater numbers of higher education providers (public, private & corporate) competing for student enrolment and resources.

• Increased global competition for top students, especially with availability of distance education opportunities and virtual universities.

Similar issues were identified in more details and more hands-on experience when individual faculties in the medical sector carried out their self-studies to perform an assessment of the current status in their pursuit for accreditation. In these studies, some of the faculties have adopted the WFME standards in basic medical education as their reference standards [5]. The most important problems/needs identified by these colleges include:

• The undergraduate curricula need review and reform [6-9].

• There is a need for introducing new courses based on recent advances in science, and on identified community needs [9,10].

• Faculty members need more training in curriculum planning, teaching and assessment methods, and other aspects of medical education [6,7,11].

• The process of resource allocation should be adequately revised in order to ensure enough resources for educational reform[6,8,9].

• Substantial resources have to be allocated to upgrade libraries and ICT facilities with an emphasis on computer-assisted learning [7,9,11].

• There is a marked deficiency of lab equipment due to insufficient funds, at both undergraduate and research levels [8,10,11].

• The educational program needs comprehensive repeated evaluation in order to ensure continuous improvement and self correction [6,7,11].

• The total budget allocated to research activities is very limited with no contribution from private sectors in research budget [6,10].

• There is no complete updated database for faculty contributions in scientific conferences, and for their publications in conferences and periodicals [6,7].

• Capacity building and developmental programs for non-academic administrative staff are lacking [8,9,11].

• Many clinical departments have no skills labs, e.g., surgery, emergency [8].

• There is a need for practical training of students on use of IT in self-learning, accessing information, etc [11].

• Undergraduate students are seldom involved in research [7-9].

• Didactic learning dominates clinical training, practical skill development, self learning and problem solving [6,7,9].

### Higher education enhancement projects (HEEP)

Higher education reform in Egypt started in the early 1980s. However serious budget constraints hampered achieving the objectives of that reform [12]. More recently, a strategy for education reform was established, and the reform agenda was influenced by the National Conference on Higher Education, held in February 2000. Its major aim was to redress Egypt's need to upgrade educational quality in the university sector [13].

In April 2002, The World Bank approved a US$ 50 million loan to support Egypt's initiative to improve the higher education system in the country through Higher Education Enhancement Project (HEEP). This project is part of the comprehensive strategy for education reform in Egypt [13] The HEEP focuses on three central areas: a) improving the efficiency through the reform of governance and management of the higher education system; b) improving the quality and relevance of university education to respond to the needs for new learning technologies, equipment, and human resource development; and c) improving quality and relevance of mid-level technical education [14].

The HEEP six priority projects are Higher Education Enhancement Project Fund (HEEPF), Information and Communications Technology Project (ICTP), Egyptian Technical Colleges Project (ETCP), Faculty of Education Project (FOEP), Faculty Leaders Development Project (FLDP), Quality Assurance, and Accreditation Project (QAAP) [15]. The medical sector faculties submitted proposals in mainly two out of the six projects of the HEEP, namely HEEPF and QAAP.

### The Higher Education Enhancement Project Fund (HEEPF) in the medical sector

The HEEPF started in 2003 aiming at assuring the competitive potentiality of higher education institutions and supporting decentralization and administrative autonomy in order to achieve progress in quality, efficiency, and effectiveness in higher education systems and institutions. The objectives of the HEEPF are: a) creating a competitive atmosphere that helps in the development of higher education institutions (departments/faculties/universities); b) encouraging decentralization and autonomy of educational institutions and the continuous self development of the educational process; c) enhancing the potentials of academic institutions for the development and establishment of modern scientific specializations; d) strengthening the cooperation and integration between industry and higher education institutions; e) developing Management Information Systems (MIS); and f) increasing the sources of information, preparing laboratories, and organizing their usage [15].

**Figure 2:**
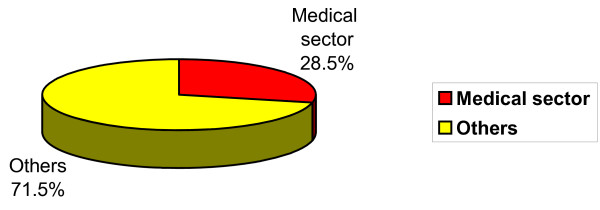
Share of the medical sector projects in HEEPF.

HEEPF financed 158 projects in different faculties and institutions of the public secular universities with a total budget of US$13 million. The share of the medical sector, in the four cycles of the project, was 45 (28.5%) out of the 158 projects (see Figure 2). They are distributed as following; 33 for medicine, 3 for pharmacy, 3 for dentistry and 6 for nursing schools. Thus, it is clear that the medical sector has a large stake in these competitive projects (45 projects for 49 colleges). Within the medical sector, colleges of medicine have got the lion's share (33 projects for 14 colleges) [15]. This might reflect the strong feelings of the medical staff regarding the need for reform.

#### Outcomes of HEEPF projects

The broad areas targeted by HEEPF projects in the medical sector and the numbers of projects in each area are shown in Table 1. It is obvious that almost all projects are not limited to only one area, but are rather targeting several areas at the same time. This comprehensive approach in facing various aspects of problems at the same time is more effective than dealing with each separately.

**Table 1:** Areas targeted by HEEPF projects in the medical sector

Targeted areas*	**Number of projects (n = 45)**	
	
	No.	%
Human resource development	44	97.8
Curriculum/course development	20	44.4
Computer-assisted learning	15	33.3
New teaching techniques-methodologies	14	31.1
Establishing training and specialized centers	14	31.1
Information technology	6	13.3
Knowledge resources	4	8.9
Quality assurance and management	4	8.9
Establishing labs/museums/virtual Labs	4	8.9
New programs	2	4.4

(*) Not mutually exclusive

The area of human resources development was addressed in most of the projects (97.8%). This shows how projects' managers value the human factor in any developmental process, and recognize it as a principal component in their plans. The target population (human resources) involved students, faculty members, administrators and/or technicians. This comprehensiveness reiterates that achievement of any progress is not dependent on a single professional category of people but it is a joint effort of all categories of workers in the medical sector.

Slightly less than half of the projects (44.4%) were concerned with developing courses or curricula for the undergraduates or postgraduates. These were aiming at improving the quality of education by fostering innovative approaches like community-based education, primary care, problem-solving skills, evidence-based medicine, and research methodology. Some of them concentrated on technical and administrative aspects. Others emphasized the practical and clinical skills, hospital safety measures, emergency and first aid measures, instrumental analysis, and clinical pharmacy.

Computer assisted learning and computer aided teaching techniques were emphasized in one third (33.3%) of the projects. All five specialties of the medical sector participated in this area. The developed learning materials varied between electronic animated teaching courses, electronic interactive instructional modules, online courses and electronic books, imaging archives, computer-based evaluation forms, video conference for live transmission of surgical procedures and scientific events, multimedia video-films for different clinical skills, and electronic media for analytical problems [16].

New teaching techniques/methodologies, including new assessment tools, were developed by slightly less than one third (31.1%) of the medical sector projects. The new methodologies were stressing intellectual, presentation, clinical, surgical, computer skills, and clinical competence. Knowledge and attitude learning domains were taken care of in some of the developed teaching tools [17].

Training and specialized centres were established by 31.1% of the medical sector projects in different fields. These included information technology, X-ray, infection control, disaster medicine, medical education, evidence-based medicine, life-saving and life support, maintenance of medical equipment, early diagnosis of disabilities, endoscopic surgery, burn management, and dental care. All the established centres are offering training and education for undergraduates and postgraduates, and/or service. Collaboration between institutions and community non-governmental organizations (NGOs) was achieved in those centres [17].

The area of information technology (IT) was tackled by 13.3% of the projects. They applied IT in establishing database and electronic libraries for labs, departments, hospitals, or setting up network connections between different places inside or outside institutions [15].

One of the principles of learning is elaboration on information [18], which means obtaining information from different resources. This principle was achieved through providing different knowledge resources by some projects, mainly in the medicine specialty of the medical sector (8.9%)

Although QAAP is one of the six projects of the HEEP, 8.9% of the medical sector projects of the HEEPF addressed the area of quality assurance and management to get ready for accreditation. Those projects were mainly conducted by medicine and nursing colleges. This important area is supposed to be addressed by almost all schools in Egypt, later on, through the QAAP.

Establishing labs, museums and virtual labs was targeted by 8.9% of the projects. The aims of those labs were enhancing research, teaching large numbers of students, performing surgical procedures, in addition to analytical processes. Introduction of new programs was done by only two projects (4.4%). These were also in the medicine specialty, and both were for postgraduates. Their fields were medical education and community medicine, which are very important and demanding fields.

Additional outcomes for the medical sector projects were; developing websites, publications, establishing computer labs and building bridges with other institutions through integration programs [15].

#### Integration of the HEEPF projects

One of the main objectives of HEEPF is strengthening cooperation among projects of institutes of higher education. Hence, integration was fostered to achieve maximum collaboration between projects in the same specialty (horizontal integration) and between projects in the same faculty and/or university (vertical integration).

The objectives of the integration in the medical sector were clear-cut: a) acquaintance and awareness among the different projects b) studying potentials and methods of integration between activities of different projects c) integrating possible dissemination and sustainability plans among projects d) Formation of a core management team with a representative from each project. Several activities were organized to achieve these objectives, including inauguration of fixed meetings, a workshop, and developing templates for integration of activities [19].

Products of this integration included a) dissemination of objectives of projects, its activities and the outcomes among participating bodies. b) exchange of knowledge and experience among faculty members of the same college, as well as, colleges of other universities. c) adding more beneficiaries d) exchange of some deliverables and resources e) creating more links and collaboration schemes among projects which assure sustainability [17].

#### Quality control of the HEEPF projects

HEEPF was keen to apply quality control measures throughout duration of projects in order to ascertain high quality of the implemented activities and their outcomes. Monitoring mechanisms included internal auditing, external auditing, as well as local and foreign peer reviewing. Examples of the tools and techniques used by medical sector projects for quality control measures include questionnaires for evaluation of the products by all beneficiaries, pre/post tests, assessment of utilization of materials, in addition to reference standards for courses or curricula. Self-evaluation reports of the management and implementation teams about their work were also submitted as quality control documents [20].

#### Assessment of performance of HEEPF projects

Regular assessment of performance of the projects was done by the HEEPF monitoring team. The criteria used for assessment were [20]:

• Outcomes are matching objectives

• Activities are done in time

• Quality of outcomes in terms of preparation, execution, cost and time

• Indicators of success were approached

• Methods of dissemination and quality control measures were taken care of

• Sustainability

• Suitability of the setting and venue of the project to the activities

• Extent of utilization of requested equipments

• Proficiency of the management team

According to the used criteria of assessment, performance of the HEEPF projects of the medical sector was shown to be very high and the projects' rating ranged between very good and excellent.

#### Sustainability of HEEPF projects

The issue of sustainability of projects is a major concern to HEEPF. The continuity of the projects in serving students, schools, universities, other institutions, and communities is one of the HEEPF main targets. Regular follow-up visits were rendered by HEEPF monitoring team to projects sites to make sure there is sustainability. Many sustainability mechanisms were achieved by the medical sector projects, which can be summarized as follows [17,20]:

• Setting up agreements with other institutions to disseminate outcomes of the projects. The medical sector was the second sector (after the science sector) among the HEEPF projects to develop those agreements. The agreements held by the medical sector projects were done with non-governmental organizations, other universities, ministry of health, syndicates, hospitals and private schools.

• Establishing self-sustained special units, which can be self-financed. The medical sector ahead of other sectors in this respect.

• Submitting proposals for other funding agencies to expand or execute another phases of the project. Some medical sector projects submitted proposals to TEMPUS (Trans-European Mobility Scheme for University Studies) and most of these have been approved.

#### Impact assessment of HEEPF projects

The overall goal of impact assessment is to identify whether HEEPF projects have efficiently achieved their planned goals and to assess its impact on the reform of higher education. The study of impact assessment of the HEEPF projects is still going on; the current phase is dealing with outcome assessment related to implementation of projects rather than long-term impact assessment [21].

#### Problems encountered during the management of HEEPF

As a part of the self assessment process of the HEEPF, some problems and weaknesses were recorded by the management team [22]; some examples are as follows:

• The lack of quantitative studies of needs assessment in most of the participating faculties, before the commencement of the projects, has deprived the selection process of an important determinant criterion.

• Insufficient coordination in the management of the HEEP big six projects resulted in some overlap between HEEPF projects' activities and other projects' activities such as QAAP.

• Lack of enthusiasm of some individual projects' managers for the required integration with the other related projects has resulted in suboptimal utilization of mutually available resources.

• The dissemination of the projects achievements and its role in educational enhancement was relatively lagging, and was not done through a well-planned system of marketing.

• Inability of some projects managers to provide sustainability plans that depend on partnership between the university resources and the private sector.

• Inability or lack of enthusiasm of some institutes to participate in HEEPF has deprived some universities from a real chance to enhance their education

### The extent to which HEEPF projects responded to the problems and challenges of higher education in the medical sector

Although the HEEPF for the medical sector did not cover all colleges, 45 projects for the 49 colleges of the medical sector is a figure which can certainly have an impact on some of the educational problems. The main and major problem identified by the medical sector, which is "constrained financial resources", has been greatly surmounted by the funding made available for these projects. This helped solving defined problems such as inadequate infrastructure, lack of modern technology, and poor instructional materials and equipment. Overcoming these problems should have an impact on the quality of education. The problem of under-trained faculty members and non-academic administrative staff was successfully addressed by almost all projects. About half of the projects responded to the problem of inadequate and insufficient courses, curricula and programs. Curriculum development and reform, of course, is a great pillar in the education reform. The problems of lack of database, computer facilities, computer-assisted materials and online learning were also dealt with by about half of the projects. This, likely, would improve the quality of teaching and might, indirectly overcome the problem of increased numbers of students. The problems of insufficient students' clinical training and lack of practical training on IT were also addressed by many projects, which would ultimately help providing the community with more proficient graduates. Although the problem of lack of accreditation mechanisms was entertained by some projects, it still needs more effort from the QAAP.

Meanwhile, some of the problems in the medical sector did not get the required priority in HEEPF projects. The problem of inadequate level of scientific research is an example. This is probably related to the complex nature of this problem, and the fact that some of its aspects are beyond the scope of the HEEPF, e.g., the limited national fund for research.

## Conclusion

The medical sector has benefited much from the HEEPF both quantitatively and qualitatively. It obtained 45 out of the total 158 projects (28.5%), with a sum total of 3 596 326 out of the 13 million US$ (27.7%) budget allotted to all projects. This is an enormous share in view of the relatively small size of this sector, which hosts only 8% of the total students enrolment within the Egyptian system of higher education. This disproportionately large contribution reflects the bad need of this sector for reform projects due to its special nature as it deals with human health, and depends greatly on practical training which requires many trained human resources and extensive infrastructure.

The projects of the medical sector have covered ten areas of education enhancement that addressed the common problems identified in the self-studies done by several colleges in this sector. The most commonly targeted areas are human resource development (97.8%), curriculum/course development (44.4%), and computer-assisted learning, together with information technology (46.6% for both together). The least commonly targeted areas were creating new programs (4.4%), new labs (8.9%), knowledge resources (8.9%), and quality assurance systems (8.9%). The areas which have been addressed in a fair frequency are developing new teaching techniques (31.1%) and establishing training centres (31.1%),

This frequency distribution of the addressed areas probably reflects the relative weight of perceiving the defined problems. Some areas were infrequently addressed in spite of their importance e.g. quality assurance, this may be due the presence of a separate project for quality (QAAP), in which all colleges in this sector would eventually participate

The projects of the medical sector as well as those in other sectors were continuously monitored, integrated with each other both vertically and horizontally, and are planned to be self-sustainable.

In order to make use of the lessons learnt in this round, the following recommendations can be concluded from this study:

1. Competitive projects represent a suitable way for educational reform as they are funnelled to the neediest sectors and the most needy colleges; thus they should remain a continuous policy in higher education reform.

2. There is a need for a quantitative assessment of the problems and challenges facing the medical sector in order to plan and evaluate the future projects accordingly.

3. Completing the study of the impact assessment of the projects and ensuring its wide dissemination should be emphasized.

4. The results of long-term follow-up of sustainability of the projects should be critically appraised, published and disseminated in order to improve sustainability in future rounds.

5. Colleges should be encouraged to involve more stakeholders such as non-governmental organizations and industrial sector in these competitive projects.

6. Allowing marketing of the tangible products of the projects among different governmental institutions as a means to support the sustainability is strongly recommended and will constitute a moral incentive for the colleges and institutions.

7. Encouraging the institutions which did not share in the first round to participate in the next one through technical support by a team of experts.

8. Involving the public religious (Al Azhar) body in the HEEPF is suggested as it is supported by the government and suffers from similar problems of the medical sector.

9. Involving the private body in partnership with the public body in conjoint projects can ensure a high quality of education in this private body, which represents 13.7% of the medical sector

10. Encouraging projects that address computer-assisted learning and online education.

11. Special projects should be planned by the HEEPF to boost research activities.

## Competing interests

The authors declare that they have no competing interests.
